# Anticipative tracking in two-dimensional continuous attractor neural networks

**DOI:** 10.1186/1471-2202-16-S1-P288

**Published:** 2015-12-18

**Authors:** Yuanyuan Mi, Yan Xia, Qi Gao, Si Wu

**Affiliations:** 1 State Key Laboratory of Cognitive Neuroscience & Learning, Beijing Normal University, Beijing 100875, China; 2 Department of Neurobiology, Weizmann Institute of Science, Rehovot 76100, Israel

## 

Time delays exist pervasively in neural information processing. The brain needs to compensate for these delays in order to extract motion information in time [1]. Experimental data reveal that in tracking moving stimuli, neural systems generate anticipative responses to compensate for delays [2], but how does the brain achieves this goal remains poorly understood.

Continuous attractor neural networks (CANNs) are widely used as a canonic model to describe the encoding of continuous features, such as head-direction, moving direction, orientation or spatial location of an object, in the brain. A CANN holds a continuous family of localized stationary states, called bumps. These bumps form a sub-manifold in the network state space, on which the system is neutrally stable, and this neutral stability endows the network with the capacity of tracking moving inputs smoothly [3]. Although a CANN is able to track a moving input, its reaction time is always delayed with respect to the instant position of the input, due to that neurons responding to external input and neuronal interaction via recurrent synapses consume time.

In this study, by incorporating spike frequency adaptation (SFA), a negative feedback mechanism, in the network dynamics, we find that a CANN is able to track a moving input anticipatively. We consider a two-dimensional CANN, mimicking the neural circuit of place cells [4]. With theoretical analysis and numerical simulation, we systematically explore the dynamics of a 2D CANN, and find that: 1) in the absence of SFA or in the presence of weak SFA, the CANN holds static bump states; 2) when SFA is sufficiently strong, the CANN generates a traveling wave, in which a bump moves spontaneously in the network without relying on external drive. The speed of the traveling wave is fully determined by the network parameters, which we call the intrinsic speed *v*_int _of the network; 3) in response to a moving input with speed *v*_ext_, the interplay between the intrinsic mobility of the network and the speed of the external drive determines the network tracking performance, that is, a) when *v*_ext_*> v*_int_, the network state lags behind the external input; and b) when *v*_ext_*< v*_int_, the network state leads the external input. Interestingly, we find that the anticipative time of the network, given by *s/v*_ext _with *s *the leading distance, is approximately a constant for a wide range of input speed. This property justifies the experimental finding that in a given brain area, the anticipative time is approximately a constant independent of input speed. By constructing a hierarchical model with multiple CANNs, our model also reproduces the experimental finding that along the signal pathway, the anticipative time of neurons increases.

Our study reveals that a neural system can utilize the intrinsic dynamics of a neural circuit to implement anticipative responses to moving inputs. It sheds light on our understanding of how the brain processes motional information in a timely manner.
